# Prognostic value of genomic mutation signature associated with immune microenvironment in southern Chinese patients with esophageal squamous cell carcinoma

**DOI:** 10.1007/s00262-024-03725-2

**Published:** 2024-06-04

**Authors:** Yue Zhou, Li Chu, Shuyan Li, Xiao Chu, Jianjiao Ni, Shanshan Jiang, Yechun Pang, Danru Zheng, Yujuan Lu, Fangcen Lan, Xiuyu Cai, Xi Yang, Zhengfei Zhu

**Affiliations:** 1 Department of Radiation Oncology, Fudan University Shanghai Cancer Center, 270 Dong An Road, Shanghai, China; 2 Department of Oncology, Shanghai Medical College, Fudan University, Shanghai, China; 3 Department of Radiation Oncology, Ruijin Hospital, Shanghai Jiaotong University School of Medicine, Shanghai, China; 4 Department of VIP Inpatient, Sun Yet-Sen University Cancer Center, State Key Laboratory of Oncology in South China, Guangzhou, China; 5 Department of VIP Inpatient, Sun Yet-Sen University Cancer Center, Guangdong Provincial Clinical Research Center for Cancer, State Key Laboratory of Oncology in South China, Collaborative Innovation Center for Cancer Medicine, 651 Dongfeng Road East, Guangzhou, China; 6 Institute of Thoracic Oncology, Fudan University, Shanghai, China

**Keywords:** Esophageal squamous cell cancer (ESCC), Genomic mutation, Risk model, Infiltration immune cells

## Abstract

The genomic landscape of esophageal squamous cell cancer (ESCC), as well as its impact on the regulation of immune microenvironment, is not well understood. Thus, tumor samples from 92 patients were collected from two centers and subjected to targeted-gene sequencing. We identified frequently mutated genes, including TP53, KMT2C, KMT2D, LRP1B, and FAT1. The most frequent mutation sites were ALOX12B (c.1565C > *T*), SLX4 (c.2786C > *T*), LRIG1 (c.746A > *G*), and SPEN (c.6915_6917del) (6.5%). Pathway analysis revealed dysregulation of cell cycle regulation, epigenetic regulation, PI3K/AKT signaling, and NOTCH signaling. A 17-mutated gene-related risk model was constructed using random survival forest analysis and showed significant prognostic value in both our cohort and the validation cohort. Based on the Estimation of Stromal and Immune cells in Malignant Tumor tissues using Expression (ESTIMATE) algorithm, the Tumor Immune Dysfunction and Exclusion (TIDE) algorithm, and the MCPcounter algorithm, we found that the risk score calculated by the risk model was significantly correlated with stimulatory immune checkpoints (TNFSF4, ITGB2, CXCL10, CXCL9, and BTN3A1; *p* < 0.05). Additionally, it was significantly associated with markers that are important in predicting response to immunotherapy (CD274, IFNG, and TAMM2; *p* < 0.05). Furthermore, the results of immunofluorescence double staining showed that patients with high risk scores had a significantly higher level of M2 macrophage than those with low risk scores (*p* < 0.05). In conclusion, our study provides insights into the genomic landscape of ESCC and highlights the prognostic value of a genomic mutation signature associated with the immune microenvironment in southern Chinese patients with ESCC.

**Supplementary Information:**

The online version contains supplementary material available at 10.1007/s00262-024-03725-2.

## Background

Esophageal cancer is a highly aggressive cancer, ranking seventh in terms of incidence and sixth in mortality worldwide, with a 5-year survival rate of 20–30% after curative surgery [1, 2]. Esophageal squamous cell cancer (ESCC), the predominant type of esophageal cancer in east Asia, is the fourth most frequently diagnosed cancer and the sixth leading cause of cancer-related deaths in China [3, 4]. The major risk factors for ESCC are not fully understood, as environmental exposure factors such as tobacco smoking and alcohol consumption only account for 46% of ESCC incidence and mortality in China [5]. But in the USA, these factors account for more than 90% of risks [6]. Sequencing of ESCC samples from US cases and Chinese cases has pinpointed several recurrently mutated ESCC-relevant genes, which suggested that ESCC cases from different geographic regions might differ genetically [7]. We sought to define the mutational landscape of ESCC in a larger cohort of southern Chinese cases to identify additional significantly mutated genes and potential prognostic markers for ESCC.

Whole exome sequencing or targeted-gene sequencing (TGS) technology is particularly useful for studying complex genomic changes, and it has been applied to various malignancies including ESCC [8–10]. Large-scale whole exome sequencing studies of ESCC cohorts have been conducted in China, revealing novel ESCC-associated genes such as *ZHF750*, *FAT1*, *FAT2*, *ADAM29*, and *FAM135B*, in addition to well-known cancer-related genes like *TP53*, *PIK3CA*, and *CDKN2A* [11, 12]. However, the prognostic implications of these somatic alterations in ESCC are not well understood. Further understanding of these genetic mutations is crucial for selecting prognostic genetic biomarkers, identifying high-risk ESCC patients with relevant genetic mutations, and tailoring treatment strategies in clinical practice.

In recent years, immune checkpoint inhibitors have shown promising therapeutic efficacy in locally advanced solid tumors beyond ESCC when combined with chemoradiotherapy with programmed cell death 1 (PD-1) blockade as first-line treatment [13, 14]. However, mono- or combined immune checkpoint inhibitors have limited effects in some patients with ESCC [15]. To improve the clinical application of immune checkpoint inhibitors, a better understanding of the tumor microenvironment of ESCC is needed.

Herein, we performed TGS of 92 ESCC tumors and adjacent normal tissue from individuals recruited from two centers in China. We compared our data with those of ESCC samples in the International Cancer Genome Consortium (ICGC) to extract the mutational signatures that cause somatic mutations in ESCC, identified driver genes or pathways contributing to the high incidence in southern Chinese ESCC, and established a reliable risk model based on mutated gene signature for predicting survival. Furthermore, we found that the risk model can potentially discriminate immune cell infiltration among patients with different risk scores.

## Methods

### Study population

We retrospectively identified patients who underwent surgical resection of the esophagus and were histologically confirmed ESCC at Fudan University Shanghai Cancer Center (FUSCC) and Sun Yet-Sen University Cancer Center (SYSUCC) from 2007 to 2008. After obtaining informed consent, cases were interviewed to collect information on demographics and clinical information. Exclusion criteria included missing baseline clinicopathological features, mixed histology and incomplete follow-up data. This study was conducted in accordance with the provisions of the Declaration of Helsinki and was approved by Institutional Review Boards of FUSCC and SYSUCC.

Data on somatic mutation, and/or the mRNA expression data, and the corresponding clinicopathological characteristics of ESCC patients from the ICGC database (https://dcc.icgc.org/) and The Cancer Genome Atlas (TCGA) database (https://portal.gdc.cancer.gov/) were acquired to make comparative analysis. Clinical characteristics included age, TNM stage, survival status, and survival time.

### Biological specimen collection and processing

Tumor tissues obtained during surgery were formalin-fixed, paraffin-embedded (FFPE), and stored at -130℃ until used. The specimens were chosen for this study based on two criteria: (a) histological diagnosis of ESCC independently confirmed by two experienced pathologists at FUSCC and SYSUCC; (b) availability of purity tumor tissue (at least > 75%). To address the FFPE-DNA damage, mitigation strategies were used in the process of sample quality control, DNA repair treatments, and analytical sample preparation, as suggested by previous studies [16, 17].

Genomic DNA was prepared for paired-end library construction using a QIAamp DNA FFPE Tissue Kit and QIAamp DNA Blood Midi Kit (Qiagen, Hilden, Germany). FFPE-induced sequencing artifacts were controlled mainly through the following aspects. Firstly, for DNA extraction, a FFPE sample underwent a pathologist review to ensure each sample at least had the area of one square centimeter, nucleated cellularity of 80% and tumor content of 20%. And specialized FFPE sample reagents were utilized for DNA extraction. Secondly, data quality was inspected and controlled by examining sequencing coverage and uniformity. The variation of CT/GA was eliminated if the ratio of transition and transversion was too high.

### Targeted-gene sequencing

The genomic alterations were examined using the YuanSuTM panel (OrigiMed, Shanghai). This panel covers 637 genes important in the oncogenesis of solid tumors. The gene list is available upon request. The targeted regions of YuanSuTM panel were captured and sequenced at a mean depth of 1000× with an Illumina NovaSeq 6000. The details of genomic alteration detection including copy number variations (CNVs) were listed in the supplemental materials.

### Tumor mutation burden (TMB) values of patients with ESCC

TMB was estimated by counting the coding somatic mutations, including single-nucleotide variants (SNVs) and insertions or deletions (INDELs), per megabase of the sequence examined in each patient. After calculating the TMB of each patient in this method, we divided the patients into high- and low-TMB groups according to the median or the quantile value for subsequent analyses.

### Protein–protein interaction (PPI) network and functional enrichment pathway analysis

The top 50 most frequently mutated genes were selected and inputted into the GeneMANIA website (http://genemania.org) to generate the PPI network. The “org. Hs.eg.db,” “ggplot2,” “clusterProfiler,” and “enrichplot” packages were utilized for analysis of the Kyoto Gene and Genome Encyclopedia (KEGG) and gene ontology (GO) pathways; FDR < 0.05 was considered to be statistically significant. Immune-related gene set enrichment analysis (GSEA) of top 200 mutated genes was used to visualize the different biological processes in different groups. Gene sets with |NES|> 1, NOM *p* < 0.05, and FDR *q* < 0.25 were considered to be statistically significant.

### Construction, evaluation and validation of the prognostic model

We randomized our ESCC cohort in a 7:3 ratio, with 70% of the dataset as the training set and 30% as the validation set. In addition, ICGC ESCC dataset was used as our independent validation sets. Clinical characteristics and gene mutation status were used as candidate features. The important features were selected by minimal depth method and predicted in the validation group. To evaluate the model, receiver operating characteristic curves were drawn and the areas under the curves (AUCs) were calculated. To evaluate the clinical significance of the selected features, we further conducted Cox regression analysis. A nomogram was constructed to show the results of Cox analysis. The R packages used in this procedure included randomForestSRC, glmnet, dplyr, survival, surviminer, ggplot2, forestplot, survcopm, and prodlim.

### Immune-related analysis

Immune cell abundance (immune score), stromal cell infiltrating level (stromal score), and tumor purity (ESTIMATE score) were estimated via the Estimation of Stromal and Immune cells in Malignant Tumor tissues using Expression (ESTIMATE) algorithm [18]. Potential response of immune checkpoint blockade therapy was estimated with Tumor Immune Dysfunction and Exclusion (TIDE) algorithm (http://tide.dfci.harvard.edu/) [19]. Using Tumor Immune Estimation Resource 2.0 (TIMER 2.0, http://timer.cistrome.org/) [20], a comprehensive analysis of immune infiltration in the tumor microenvironment of ESCC was carried out. MCPcounter algorithm was used to estimate the relative proportions of 22 immune cells in ESCC. The infiltration of 22 immune cells was indicated by enrichment scores, which were calculated by single sample GSEA using Gene Set Variation Analysis R package.

### Tissue microarrays construction and immunofluorescence double staining

Two different FFPE blocks of tumor tissues were obtained from each patient and spotted in adjacent 2.5-mm cores as per the tissue microarray map to capture the tumor heterogeneity. Briefly, the regions of interest were marked by experienced pathologists, and then a recipient paraffin block was made, incubated, cooled, fixed, and finally stored with paraffin. The details of the construction process were listed in the supplemental materials. A panel of two markers was used to explore the macrophage infiltration in ESCC, which included (a) marker of epithelial cells and tumor cells: cytokeration; and (b) marker of macrophages: CD68. A panel of two markers was further used to explore the infiltration of different types of macrophages, which included (a) marker of pro-inflammatory M1 macrophages, CD86; and (b) marker of anti-inflammatory M2 macrophages, CD163. The details of the staining process were listed in the supplemental materials.

### Statistical analysis

All statistical analyses were conducted using SPSS 24.0, R software (version 4.0.2), and GraphPad Prism 8.0. Student’s *t* test was used to compare continuous variables, and the Chi-square test or Fisher’s exact test was used to compare categorical data. Fisher’s exact test was used to analyze the correlations between TMB and gene mutations that co-occurred or were exclusive of each other. Cox regression analysis was used to determine risk mutated genes. The clinical effects of the prognostic model were assessed using the log-rank test and Kaplan–Meier method. A nomogram model was then constructed, and predictive performance of the nomogram was estimated using C-index and calibration plot.

## Results

### Patient, sample and study design

**Fig. 1:**
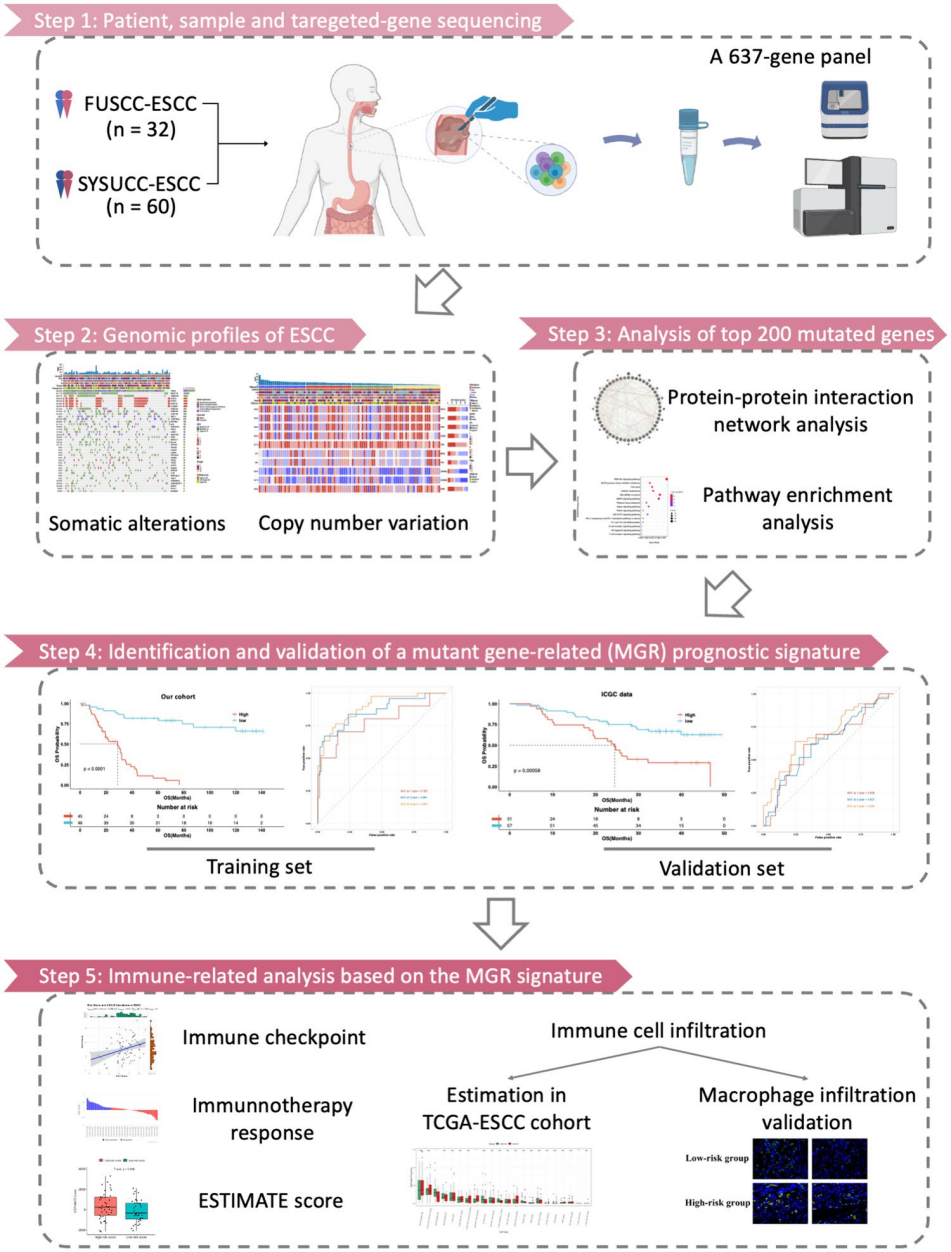
Flowchart of the study design

Primary tumor tissue samples were obtained from 102 patients with histologically confirmed ESCC. After discarding samples that failed to pass the quality check, a final cohort of 92 patients was available for analysis, with 32 from FUSCC and 60 from SYSUCC. All the patients had TGS data and CNV data. Figure 1 demonstrates the study design. The detailed clinicopathological information was shown in Supplemental Table 1.

### Landscape of genomic profiles of ESCC

**Fig. 2:**
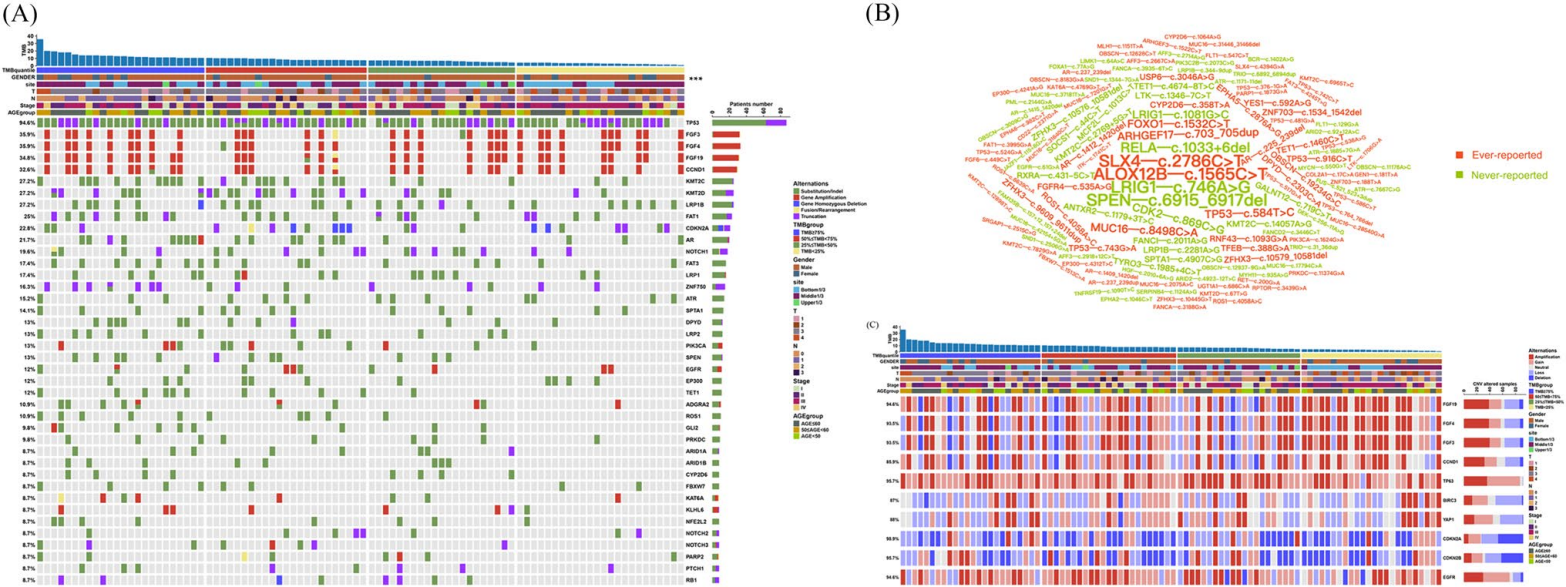
Genomic landscape of ESCC in 92 samples. **A**. Mutational landscape of somatic alterations. Top 40 frequently mutated genes are ordered by frequency. Samples are ordered by TMB values. Each column denotes an individual tumor, and each row represents a gene. Very top, TMB values (y axis) for each sample (*x* axis). Top, key clinical parameters of each examined case. Left, percentage of mutation in 92 ESCC samples. Right, vertical axis represents total number of mutations for each gene. Clinical characteristics and mutation types are shown by color as indicated. **B**. Word cloud for the somatic mutation sites. The mutation sites with percentage over 3.3% (3/92) were represented. The ever-reported sites were shown in red color, while the never-reported sites were shown in green color. **C**. Mutational landscape of copy number variations (CNVs). The 10 genes with frequent CNVs are ordered by frequency. Samples are ordered by TMB values. Each column denotes an individual tumor, and each row represents a gene. Very top, TMB values (*y* axis) for each sample (*x* axis). Top, key clinical parameters of each examined case. Left, percentage of CNVs in 92 ESCC samples. Right, vertical axis represents total number of CNVs for each gene. Clinical characteristics and mutation types are shown by color as indicated

Among the 92 ESCC samples with qualified TGS data, 1,979 somatic mutations were identified, comprising 1,799 SNVs and 168 INDELs. These tumors harbored a median of 22 nonsynonymous SNVs and 2 INDELs. As demonstrated in Fig. 2A, the most prominent mutated genes were *TP53* (found in 94.6% of tumors), followed by *KMT2C* (27.2%), *KMT2D* (27.2%), *LRP1B* (27.2%) and *FAT1* (25%). As for the mutation sites, *ALOX12B* (c.1565C > T), *SLX4* (c.2786C > T), *LRIG1* (c.746A > G), and *SPEN* (c.6915_6917del) were the most frequent (6.5%), followed by *RELA* (c.1033 + 6del) at 5.4%. Some of these mutation sites have never been reported before (Fig. 2B). To assess somatic CNVs, reported oncogenes and tumor suppressor genes observed in the top rank (residual $q<1\times {10}^{-4}$) GISTIC peaks were examined (Fig. 2C). *FGF19* was the gene most frequently affected by somatic CNVs, with alterations in 94.6% of patient samples. Frequent amplifications were observed in *FGF3* (43%), *FGF4* (42%), *FGF19* (42%), *TP53* (39%), and *CCND1* (35%). Frequent deletions were observed in *CDKN2A* (42%) and *CDKN2B* (36%). Frequent losses were observed in *BIRC3* (45%) and *YAP1* (38%), and frequent gains in *EGFR* (45%). The mean TMB was 8.2 mutations per megabase (mut/Mb), and median TMB was 7.5 mut/Mb. After the 92 samples were divided into four groups by TMB quantiles, gender was the only one among the clinicopathological features to be significantly related to TMB (*p* < 0.001, Fig. 2A). A total of 12 mutated genes were significantly associated with TMB (*p* < 0.05), including *ZNF750*, *TP53*, *TET1, ROS1*, *LRP2*, *KMT2D*, *GLI2*, *AR*, *FAT1*, *SPEN*, *KMT2C*, and *DPYD* (Supplemental Fig. 1).

### Functionally aberrant pathways in ESCC

**Fig. 3:**
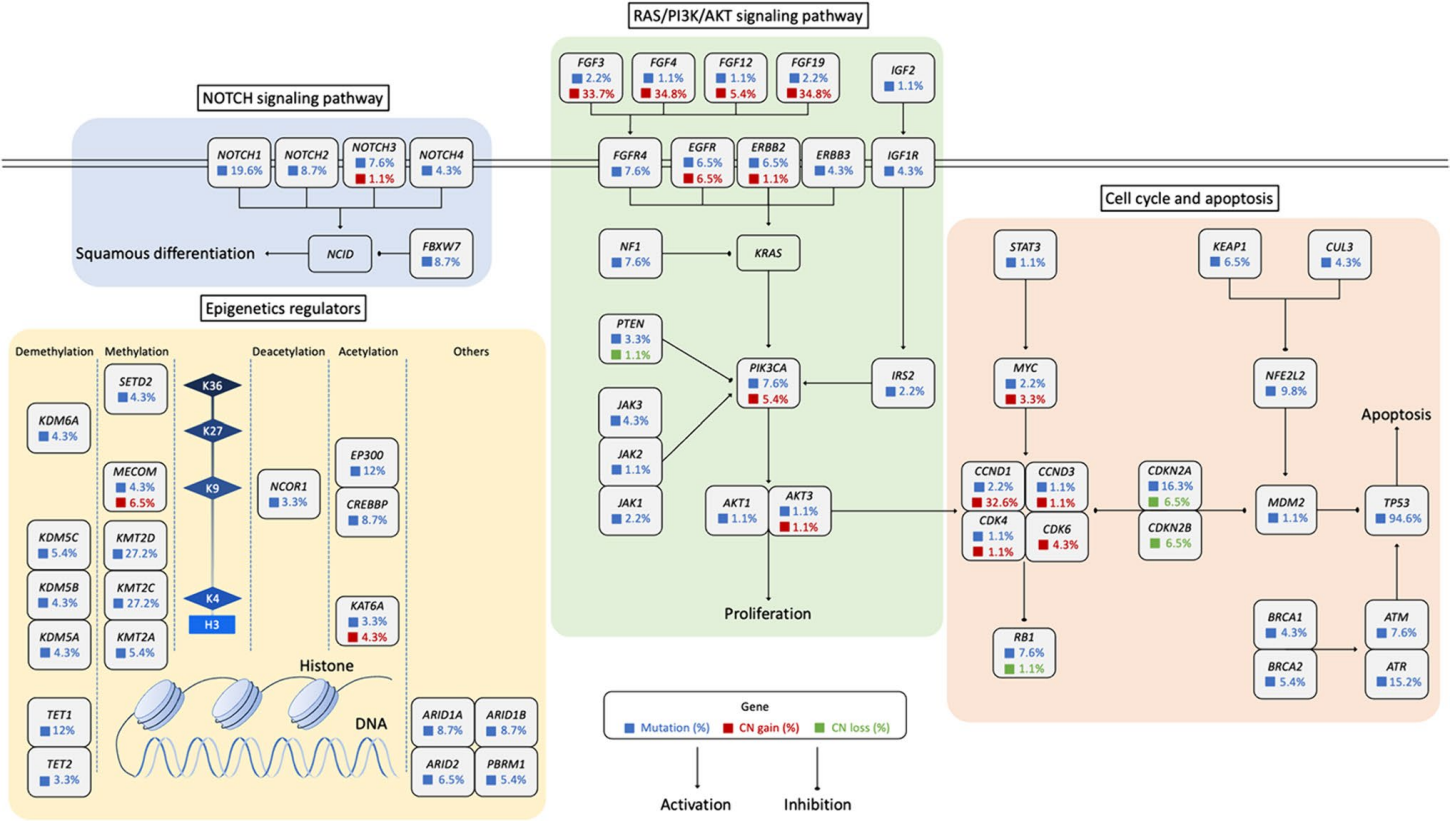
The aberrant pathways and networks in 92 ESCC samples

We integrated the top 200 most frequent mutated genes into pathway analysis to identify the functionally aberrant pathways in ESCC. As a result, we identified a highly interconnected network of aberrations targeting the cell cycle regulators, epigenetics regulators, PI3K/AKT signaling pathway, and NOTCH signaling pathway (Fig. 3). Several tyrosine kinases (that is, *FGF3*, *FGF4*, *FGF12*, and *FGF19*) and receptors (that is, *EGFR* and *ERBB2*) exhibited CN gains, as well as their downstream signal transducer *PIK3CA* and *AKT3*, together contributed to the proliferation of ESCC. The loss of *CDKN2A* and *CDKN2B*, together with the gain of *CCND1*, *CCND3*, *CDK4*, and *CDK6*, resulted in the disruption of cell cycle restriction.

**Fig. 4:**
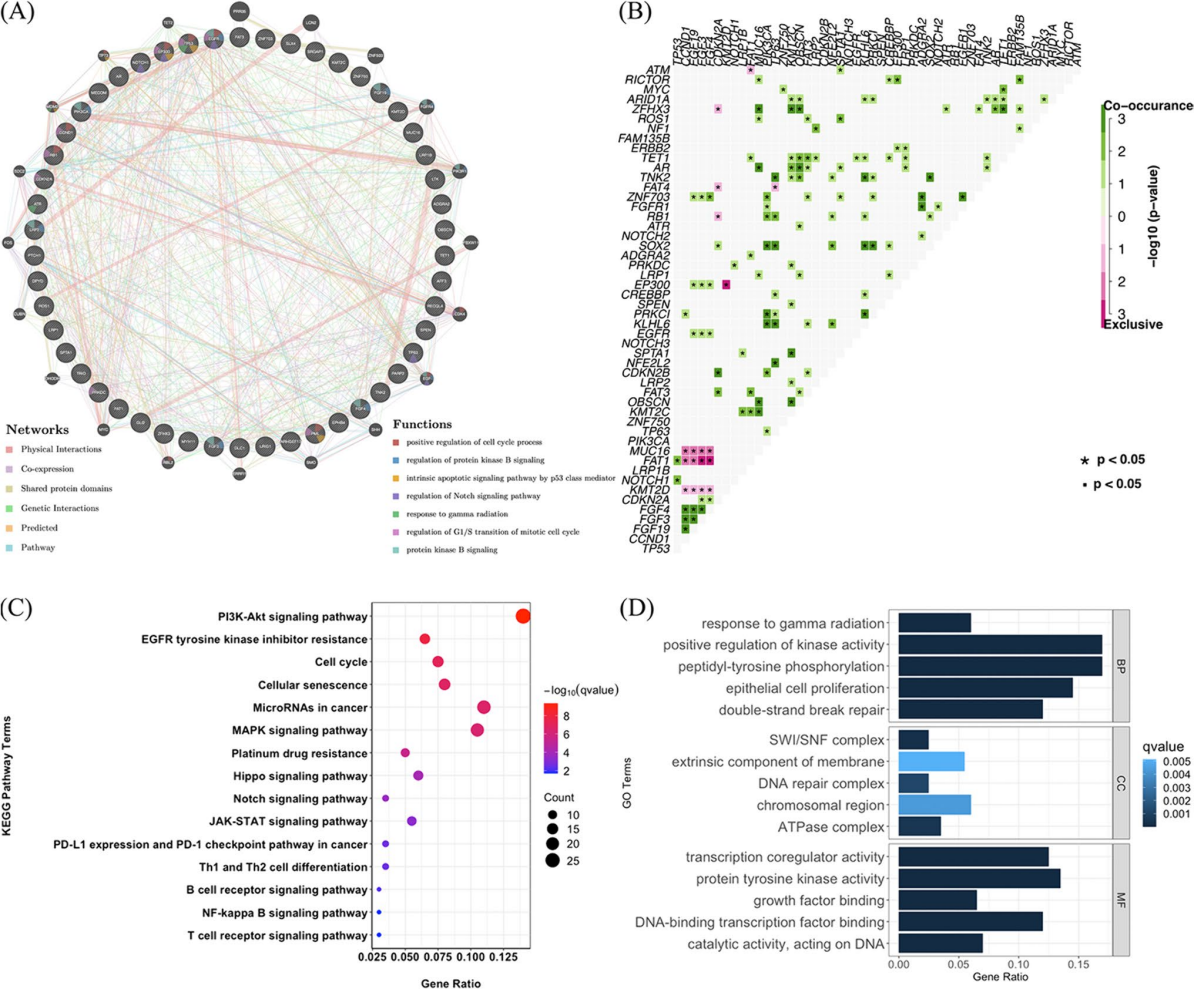
Analysis of protein–protein interaction (PPI) network, correlation, and functional pathway among mutant genes. **A**. PPI network of top 50 frequently mutated genes. Interaction types and the Gene Ontology (GO) terms are shown by color as indicated. **B**. Correlation among the top 50 frequently mutated genes in 92 ESCC samples. **C**. Top 15 enriched Kyoto Gene and Genome Encyclopedia (KEGG) pathways in The cancer genome atlas (TCGA)-ESCC cohort. **D**. Top 15 enriched GO pathways in TCGA-ESCC cohort

To determine whether the mutated genes functionally interacted with each other and were involved in tumorigenesis, the top 50 frequently mutated genes were selected to perform a PPI network-based analysis and functional pathway analysis. The PPI networks are visualized in Fig. 4A. These genes and their encoded proteins were involved in positive regulation of cell cycle process, regulation of protein kinase B signaling, intrinsic apoptotic signaling pathway, and regulation of NOTCH signaling pathway, which was similar to the results in the above aberrant pathway analysis. The co-occurrence and exclusion relationship of the mutated genes in ESCC is demonstrated in Fig. 4B. Among the top 50 frequently mutated genes, *KMT2C* and *TET1* had the most frequent co-occurrence relationship with other 13 genes in mutation, hinting the important role of epigenetic regulators in ESCC pathogenesis and development. KEGG and GO pathway analysis demonstrated enrichment of mutated genes in several signaling pathways involved in malignancy, such as the PI3K/AKT pathway, cell cycle, and NOTCH signaling pathway, all of which were also enriched in TCGA-ESCC cohort (Fig. 4C and D)

### Construction and evaluation of a 17-mutated gene-related risk model by random survival forest analysis

**Fig. 5:**
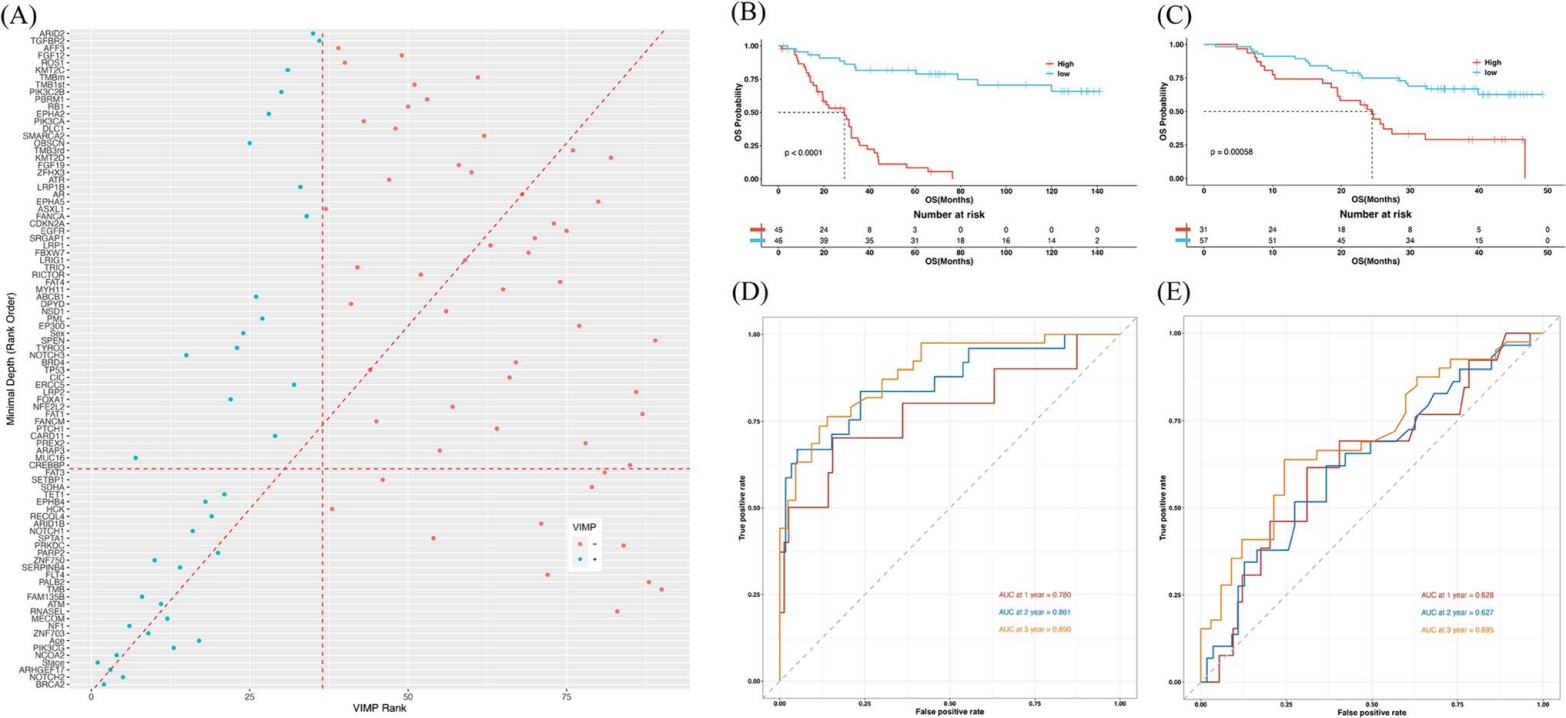
Identification and validation of a 17-mutated gene-related (17MGR) risk model. **A**. Selection of the important features by minimal depth method. Clinical characteristics and gene mutation status were used as candidate features. **B**. and **C**. Kaplan–Meier curves of the internal and external validation sets. The patients are grouped by the median value of risk score based on the 17MGR model. **D**. and **E**. Receiver operating characteristic curves of the internal and external validation sets. The patients are grouped by the median value of risk score based on the 17MGR model

**Fig. 6:**
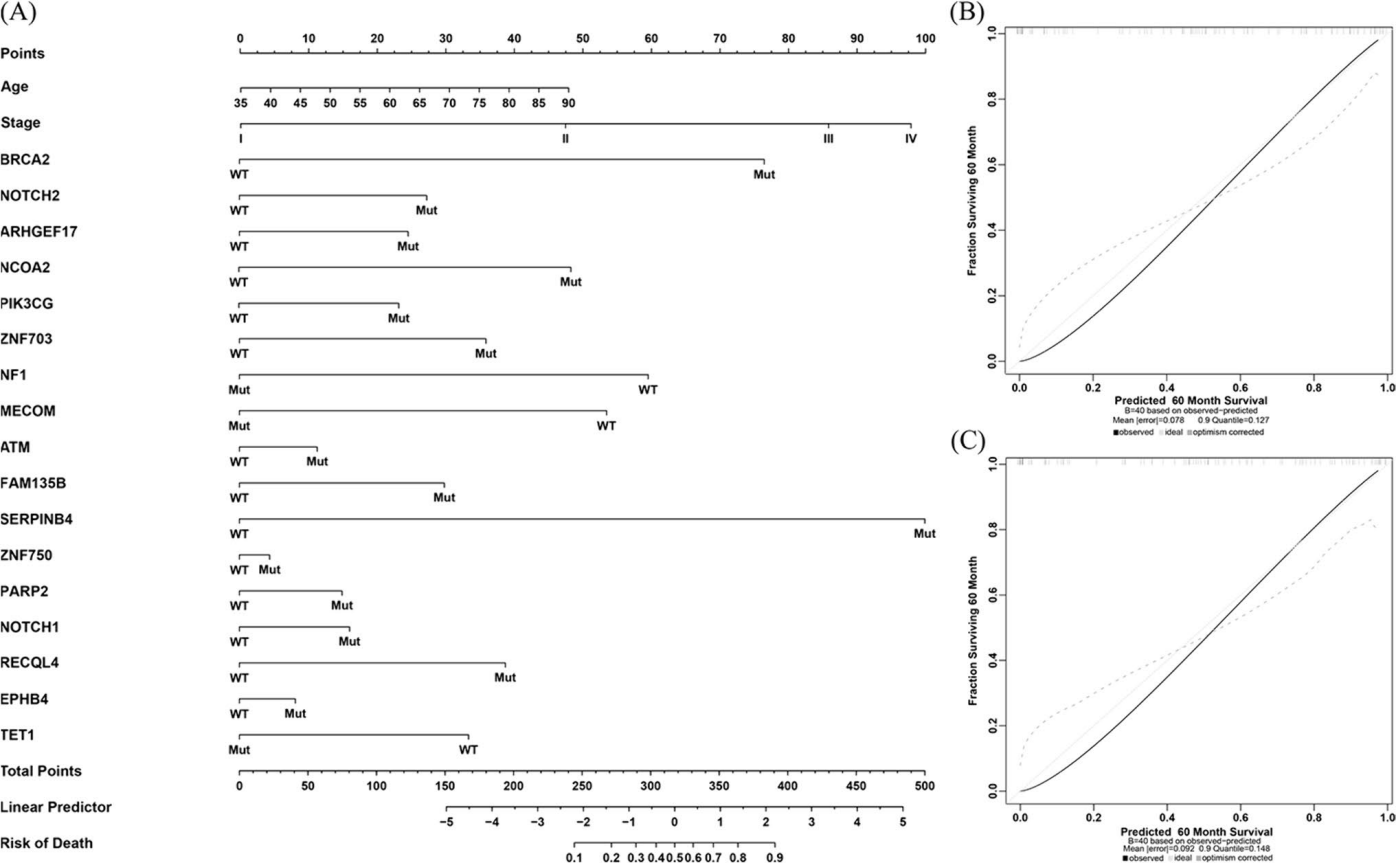
A constructed nomogram for prognosis prediction in ESCC. **A**. A constructed nomogram for overall survival prediction of a patient with ESCC. **B**. Calibration plot of the constructed nomogram in the internal validation set. **C**. Calibration plot of the constructed nomogram in the external validation set

Random survival forest model was applied to determine the mutated genes of most significance to the survival of ESCC patients. According to the variable importance and minimal depth[21], 19 variables were finally selected, including 17 mutated genes (*BRCA2*, *NOTCH2*, *ARHGEF17*, *NCOA2*, *PIK3CG*, *ZNF703*, *NF1*, *MECOM*, *ATM*, *FAM135B*, *SERPINB4*, *ZNF750*, *PARP2*, *NOTCH1*, *RECQL4*, *EPHB4*, and *TET1*), age and stage (Fig. 5A). Among the 17 genes, *NOTCH2*, *ARHGEF17*, *NCOA2*, and *ZNF750* were significantly associated with the OS of patients in our cohort (Supplemental Fig. 2). Based on the model, each patient was scored. The median risk score value was used to divide the samples into high-risk and low-risk groups both in our cohort and in the ICGC cohort. The survival curves can be significantly separated between the two groups in both cohorts (*p* < 0.001, Fig. 5B and C). To validate this model, receiver operating characteristic curves were drawn, and the AUCs for 36 months, 24 months, and 12 months were 0.890, 0.861, and 0.780, respectively in our validation set (Fig. 5D) and 0.695, 0.627, and 0.628, respectively in the ICGC validation set (Fig. 5E**)**. Then, a predictive nomogram based on these risk factors was constructed to clearly show the survival rate (Fig. 6A). Calibration curve indicated that the observed and predicted values were consistent in predicting OS in our cohort and the ICGC cohort (Fig. 6B and C).

Of note, TMB was not selected under this screening method. We further examined the association between TMB and the OS of patients in our cohort. The survival curves twined together in the patients grouped by either the median value or the quantile values of TMB (Supplemental Fig. 3).

### Immune-related analysis based on the 17-mutated gene-related risk model

**Fig. 7:**
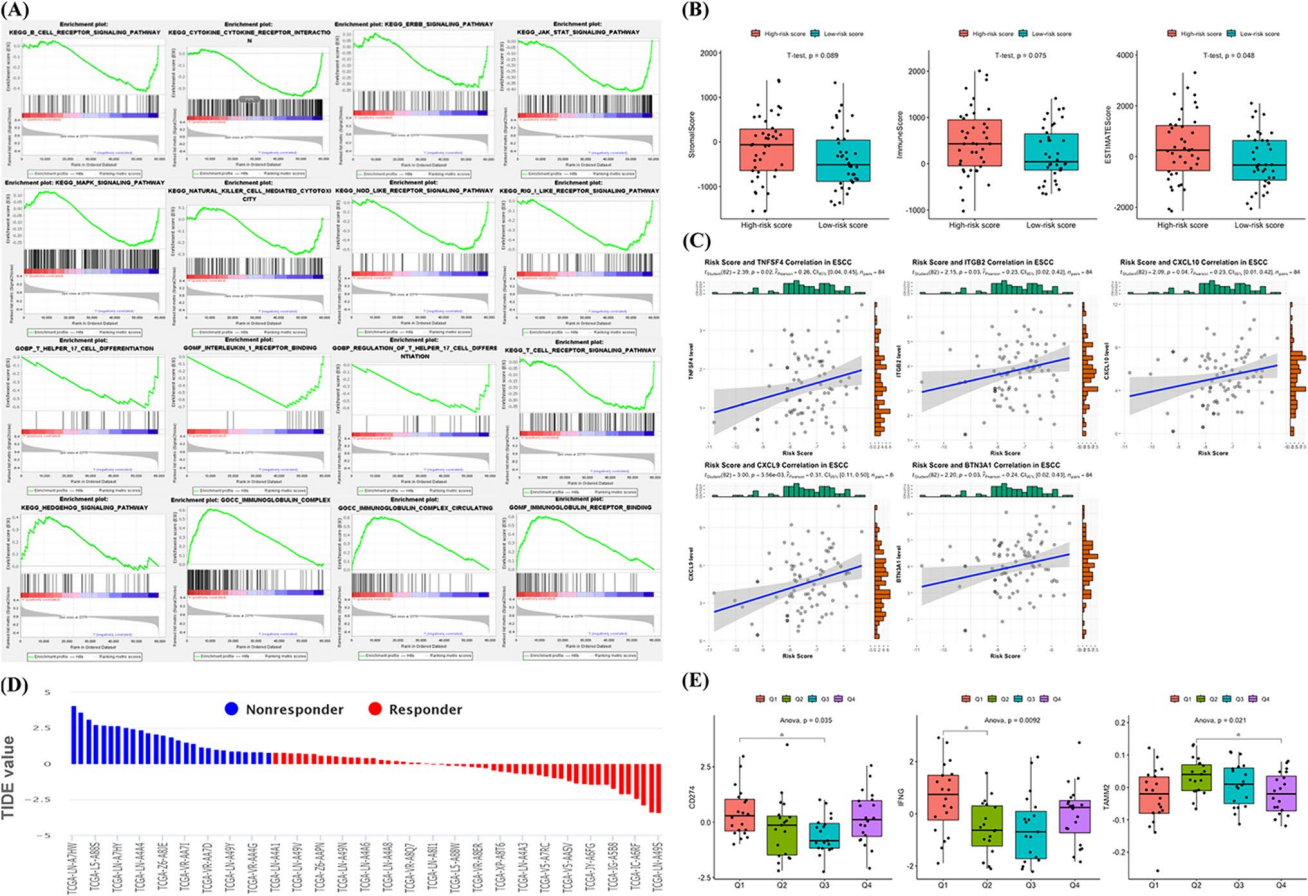
Immune-related analysis based on the MGR signature. **A**. Immune-related gene set enrichment analysis (GSEA) in the TCGA-ESCC cohort stratified by the median value of risk scores based on the 17MGR model. **B**. Stromal score, Immune score and ESTIMATE score of the TCGA-ESCC cohort stratified by the median value of risk scores based on the 17MGR model. **C**. The relationship between risk score based on the 17MGR model and the level of immune checkpoints (*p* < 0.05). **D**. The immunotherapy therapy response of the TCGA-ESCC cohort predicted by TIDE. **E**. The level of immunotherapy therapy response-related markers in the TCGA-ESCC cohort stratified by the quantile values of risk scores based on the 17MGR model

To further reveal the potential relationship between the risk score and immune microenvironment in ESCC, the TCGA-ESCC cohort with both mutation data and mRNA expression data was analyzed. We used GSEA to visualize the enriched immune-related biological processes in different risk groups classified by the mutated genes-related risk model in the TCGA-ESCC cohort. Our results indicate that patients in the low-risk group were prone to show associations with Th17 cell differentiation, regulation of Th17 cell differentiation, IL-1 receptor binding, cytokine receptor interaction, natural killer cell mediated cytotoxicity, JAK-STAT signaling pathway, B cell receptor signaling pathway, and T cell receptor signaling pathway (Fig. 7A). Compared to the low-risk group, the high-risk group was enriched in fewer immune-related pathways. Using the ESTIMATE algorithm, we found that the EstimateScore was significantly lower in the low-risk score group (Fig. 7B). The relationship between the risk score and the immune checkpoint expression level was then explored. The risk score was in a significant correlation with several stimulatory immune checkpoints (*p* < 0.05), including TNFSF4, ITGB2, CXCL10, CXCL9, and BTN3A1 (Fig. 7C). And the risk score tended to have a correlation with many stimulatory or inhibitory immune checkpoints with *p* < 0.1 (Supplemental Fig. 4). To predict the response to immunotherapy, the TIDE algorithm was used to divide the patients into responders and non-responders (Fig. 7D). The key markers in the TIDE algorithm (CD274, IFNG, and TAMM2) exhibited significantly different levels in the patients with different risk scores (Fig. 7E). Taken together, these results suggest that the 17-mutated gene-related risk model might play an additional role in hinting the immune status in ESCC.

### Immune cell infiltration analysis based on the 17-mutated gene-related risk model

**Fig. 8:**
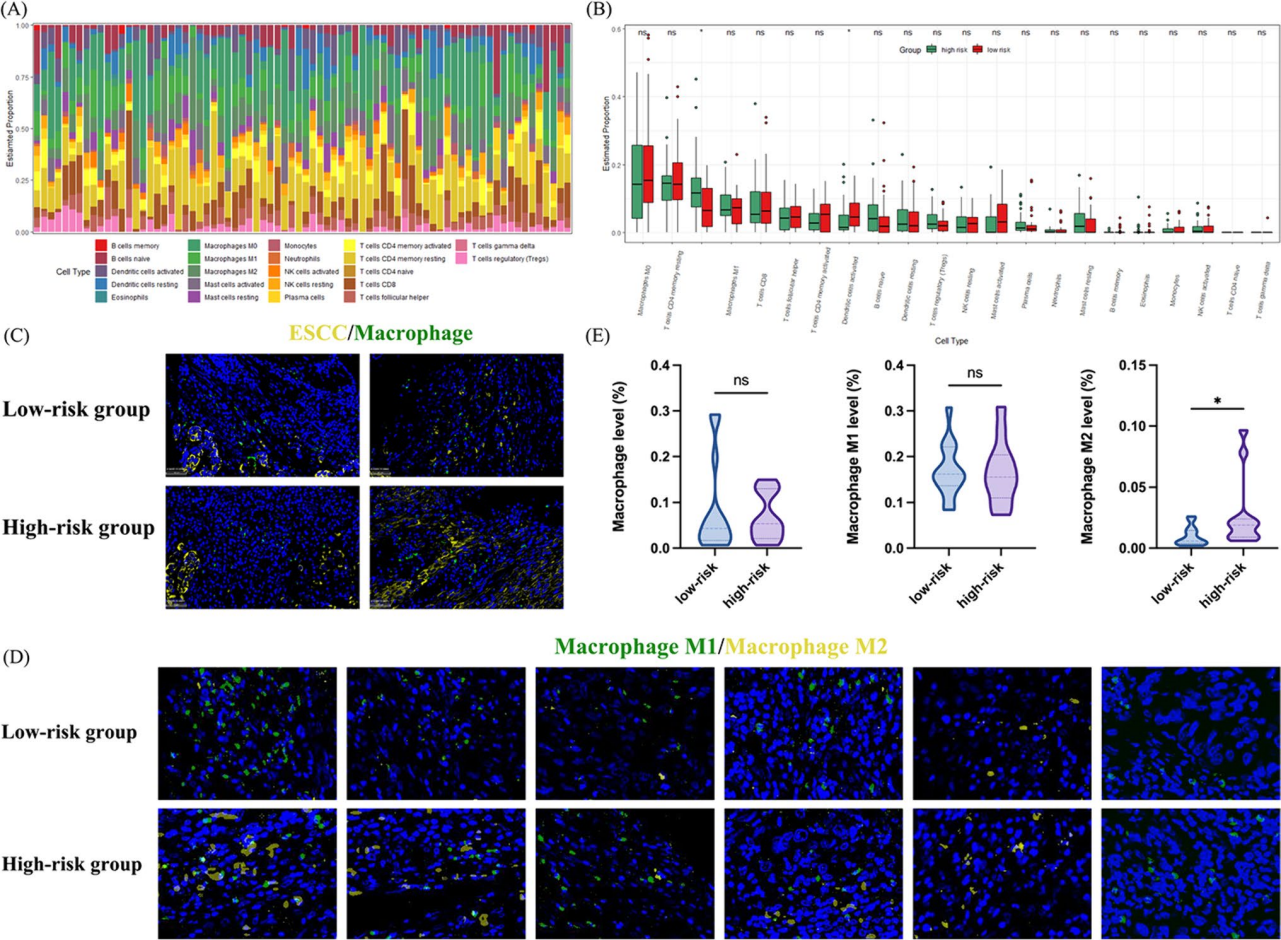
The estimation and validation of tumor-infiltrated macrophage cells in ESCC. **A**. The proportion of tumor-infiltrated immune cells in each patient in the TCGA-ESCC cohort. **B**. The level of tumor-infiltrated immune cells in the TCGA-ESCC cohort stratified by the median value of risk scores based on the MGR signature. **C**. The level of ESCC cells and tumor-infiltrated macrophages in the ESCC patients displayed by immunofluorescence double staining (*n* = 28). **D**. The level of tumor-infiltrated macrophages (M1/M2) in the ESCC patients displayed by immunofluorescence double staining (*n* = 28). **E**. The level of tumor-infiltrated macrophages (M0, M1, and M2) in the ESCC patients stratified by the median value of risk scores based on the 17MGR model

The potential relationship between the risk score and infiltrating immune cells was further explored in the TCGA-ESCC patients grouped by the median of the risk score based on the 17-mutated gene-related model. Figure 8A demonstrates the proportion of different infiltrating immune cells in each sample. Among the 22 types of immune cells, the proportion of macrophage ranked first, followed by T cells CD4 + memory resting, M2 macrophage and M1 macrophage (Supplemental Fig. 5). Compared to patients in low-risk group, patients in high-risk group were estimated to harbor a significantly higher proportion of M2 macrophage and a lower proportion of activated dendritic cells (Fig. 8B). As the proportion of macrophage ranked first, we then unveil the profile of macrophage infiltration in ESCC in our cohort. Macrophage was marked and classified as pro-inflammatory M1 macrophage and anti-inflammatory M2 macrophage by immunofluorescence double staining. Macrophage was detected in ESCC tissue from patients in both low-risk group (*n* = 14) and high-risk group (*n* = 12), with no significant difference in macrophage level (Fig. 8C and E). M1 macrophage was observed in a similar level in the two groups; however, patients in the high-risk group had a significantly higher level of M2 macrophage than patients in the low-risk group, hinting the potential role of M2 macrophage instead of M1 macrophage in the regulation of ESCC immune microenvironment (Fig. 8D and E).

## Discussion

Despite the great progress made by traditional treatment methods in ESCC, the survival rate has not shown significant improvement. Therefore, exploring the pathogenesis and biological characteristics of ESCC has become a primary focus in its treatment. To gain a deeper understanding of genomic landscape in ESCC, we conducted a comprehensive analysis of the TGS data of 92 samples collected from two centers. Our analysis revealed four main functionally aberrant pathways in ESCC. Additionally, we constructed and validated a 17-mutated gene-related risk model to provide a better prediction of prognosis. The risk model may also provide insights into the immune status of ESCC patients and aid in the development of precise treatment strategies in the era of immunotherapy.

The most frequently mutated gene identified in our study was *TP53*, followed by *KMT2C*, *KMT2D*, *LRP1B* and *FAT1*. These genes were also observed to have high-frequency mutations in other Chinese ESCC populations [22, 23]. In a Japanese population, *LRP1B* was found to be associated with recurrent focal CNVs rather than somatic mutations, suggesting different mutation types of *LRP1B* between Chinese and Japanese populations [4]. The most frequently copy number amplified and deleted genes were *FGF3*/*FGF4*/*FGF19*/*TP53*/*CCND1* and *CDKN2A*/*CDKN2B*, which is consistent with previous studies [4, 22, 24]. These CNV-affected genes may serve as potential targets for molecular-based therapies in ESCC, which needs further exploration in clinical and pre-clinical studies. Furthermore, among the 12 mutated genes associated with TMB in the current study, *KMT2D* and *SPEN* mutations have been reported to be significantly associated with high TMB and could potentially serve as prognosis biomarkers for Chinese ESCC patients [23].

Informatic analyses, including PPI network, KEGG pathway analysis, and GO pathway analysis, were performed to uncover the registry of driver genes and pathways that are somatically disrupted in Chinese ESCC. Many of the mutated genes were found to be involved in the regulation of the epigenetic pathway. Previous studies have shown that epigenetic regulators such as *EP300* and *CREBBP* can stratify patient survival [4, 24, 25]. These results suggest an important role of repressive epigenetic marks in the pathogenesis of ESCC. KEGG pathway analysis revealed that PI3K/AKT pathway, cell cycle pathway, and NOTCH signaling pathway were enriched in both our cohort and TCGA-ESCC database, indicating their importance in the development and progression of ESCC.

Several prognostic signatures or risk models have been identified in ESCC based on bioinformatic analysis using data sets from public databases like TCGA [26–28]. However, few of these studies have focused on Chinese population. In our study, we established a novel and robust risk model for ESCC patients in southern China using 17 mutated genes and two clinical factors. Although TMB has been reported as a potential independent biomarker [29], we found limited evidence of its role in predicting prognosis, which needs further exploration due to the small sample size. The constructed risk model showed satisfactory efficacy and accuracy, with AUC values of 0.890, 0.861, and 0.780 for 36 months, 24 months, and 12 months, respectively.

Despite the substantially improvements in tumor treatment brought by immunotherapy, its effects in ESCC remain limited. Our study conducted immune-related GSEA in ESCC patients with different risk scores and found that high risk was associated with fewer immune-related biological processes or pathways, which may partly explain the limited immunotherapy effect in some ESCC patients [15]. The risk score was significantly correlated with the expression level of several stimulatory immune checkpoint, and significantly associated with several markers important in predicting the response to immune checkpoint blockade therapy, suggesting the potential role of the risk model in predicting the response to immunotherapy. Additionally, high risk was associated with increased infiltration of M1 macrophages. A recent study revealed the distinct spatial distribution of PD-L1- or PD-L1 + macrophages in ESCC and found the critical importance of the close distance between tumor cells and these antigen-presenting cells to the clinical outcome in patients receiving chemoradiotherapy combined with PD-1 blockade [30]. This finding is consistent with previous pre-clinical and clinical studies that have shown a strong correlation between tumor associated macrophages and poor prognosis in ESCC patients [31–33]. Hui Yang et al. found that tumor-associated macrophages can be recruited via CCL2-CCR2 axis and induce immune evasion through PD-1 signaling in esophageal carcinogenesis [33]. M2 macrophage polarization can be promoted via different pathways, which accelerates the metastasis of ESCC, which corroborates our finding [34, 35]. All these findings suggest that as the most abundant immune cells in ESCC microenvironment, macrophages and their differentiation may be potential targets in immunotherapy in ESCC.

However, our study has some limitations. Firstly, it was based on selected cancer-related genomes, which may result in the omission of important genes or signaling pathways. Secondly, the limited sample size of ESCC in ICGC may affect the validity of the risk model. Additionally, both our data and ICGC ESCC database lack information on immunotherapy, limiting further evaluation of the clinical prognostic model in predicting the response to immunotherapy.

## Conclusions

In conclusion, our study provides a comprehensive mutational landscape of ESCC, enhancing our understanding of the molecular pathogenesis of this disease. We have established a novel and robust risk model for ESCC patients in southern China using 17 mutated genes and two clinical factors, serving as a valuable resource for the risk management. Furthermore, the risk model can provide insights into the immune landscape of ESCC patients, particularly the infiltration level of M2 macrophages, which may contribute to the development of precise treatment strategies in ESCC, especially in the era of immunotherapy.

### Supplementary Information

Below is the link to the electronic supplementary material.

Supplementary file1 (DOCX 15 KB)

Supplementary file2 (DOCX 17 KB)

Supplemental Fig. 1. The association between TMB and mutant genes (p < 0.05). (TIF 5297 KB)

Supplemental Fig. 2. The overall survival in the ESCC patients stratified by (A) the median value of TMB and (B) the quantile values of TMB (TIF 7147 KB)

Supplemental Fig. 3. Forest plot of the mutated genes in the 17MGR model associated with overall survival in ESCC (p < 0.1) (TIF 5388 KB)

Supplemental Fig. 4. The relationship between risk score based on the MGR signature and the level of stimulatory and inhibitory immune checkpoints (p < 0.1) (TIF 2970 KB)

Supplemental Fig. 5. The level of tumor-infiltrated immune cells estimated in the TCGA-ESCC cohort (arranged in descending order) (TIF 5128 KB)

## Data Availability

Datasets could be downloaded directly from the indicated websites or are available from the corresponding author on reasonable request.
