# Supplementary material for: Prognostic value of genomic mutation signature associated with immune microenvironment in southern Chinese patients with esophageal squamous cell carcinoma
Source: Cancer Immunol Immunother. 2024 Jun 4;73(8):141. doi: 10.1007/s00262-024-03725-2 (PMC11150228; doi:10.1007/s00262-024-03725-2)
Supplement: Supplementary file 1 — Supplementary file1 (DOCX 15 KB) [file 262_2024_3725_MOESM1_ESM.docx]

**Supplemental Materials**

**Genomic alteration and gene rearrangement detection**

The raw sequencing data underwent stringent quality control of read depth and ratio of target capture. Clean reads were mapped to Human Reference Genome (hg19) using BWA-MEM. Genomic alterations were identified through the following methods: MuTect (v1.17) for single nucleotide variants; PINDEL (v0.2.4) for insertion–deletion mutations (Indels), a minimum of 5 reads was required to support alternative calling. Variants with read depths less than 30× with strand bias larger than 10% or VAF < 0.5% were removed. To inspect the sensitivity of the mutation calling algorithm on sequence depth, we conducted an in silico simulation by down-sampling sequence reads using SAMtools (v1.3.1), followed by the same mutation calling steps for each down-sampled dataset. SnpEff3.0 for the functional impact of these mutations; Control-FREEC v9.4 (parameters: window = 50,000 and step = 10,000) for copy number variations, deletion and duplication events were defined on a gene-by-gene basis.

For detecting gene rearrangements, paired-end reads with abnormal insert size of over 2000 bp aligned to the same chromosome or aligned to different chromosomes were collected and used as discordant reads. The group consisting of discordant reads with a distance less than 500 bp formed a cluster and paired clusters were obtained according to the pairing relationship. Consistent breakpoints from the paired-end discordant reads within a cluster were identified to establish potential rearrangement breakpoints. The breakpoints were double confirmed by BLAT11 and the corresponding discordant reads were filtered for those uniquely mapped to the genome reference to constitute rearrangement supported reads. The resulting chimeric read candidates were genome annotated.

**Tissue microarrays (TMA) construction**

Two different paraffin-embedded (FFPE) blocks of tumor tissues were obtained from each patient and spotted in adjacent 2.5-mm cores as per the TMA map to capture the tumor heterogeneity. Initially, each block was reviewed, and the regions of interest were marked by experienced pathologists after hematoxylin and eosin staining. Subsequently, a recipient paraffin block was made with a duplicate 2.5-mm punch from each individual using the Tissue Arrayer (Beecher Instruments, Sun Prairie, WI, USA) in accordance with the positional blueprint. The recipient array blocks were incubated at 53 °C for 2 h to allow the cores to adhere to the recipient block. After cooling, the blocks were sectioned (3-µm-thick) using a microtome (Leica RM2125 RTS, Germany) and fixed on charged slides. Finally, the TMAs were finely layered with paraffin to prevent antigen decay and stored at 4 °C until use.

**Immunofluorescence double staining**

Briefly, the TMA slides were prewarmed followed by deparaffinization and then fixed in 10% neutral buffered formalin before heat-induced epitope retrieval using a preheated AR6 buffer (pH 6.0; PerkinElmer, Inc. USA). After washed with in 0.03 % tris-buffered saline–Tween-20 (TBST, Amresco) three times, the slides were sealed with 10% goat serum (S-1000, Vector Labs, Burlingame, CA) for 30 min at room temperature. Then, the sections were incubated with diluted primary antibody, namely CK (Abcam; 1/3000, 30 min), CD68 (Maxim; 1/600, 60 min), CD86 (Servicebio; 1/2000, overnight) and CD163 (Abcam; 1/200, overnight), followed by incubation with corresponding fluorescent-labelled secondary antibodies (Servicebio; 1/2000, 50 min) in the dark. Then, sections were counterstained with DAPI (D9542, Sigma-Aldrich), mounted in the Vectashield hardset® fluorescence mounting medium (Vector Labs, Burlingame, CA, USA), and imaged using the Vectra3.0 Multispectral Imaging System (PerkinElmer).
