# Supplementary material for: Prognostic value of genomic mutation signature associated with immune microenvironment in southern Chinese patients with esophageal squamous cell carcinoma
Source: Cancer Immunol Immunother. 2024 Jun 4;73(8):141. doi: 10.1007/s00262-024-03725-2 (PMC11150228; doi:10.1007/s00262-024-03725-2)
Supplement: Supplementary file 2 — Supplementary file2 (DOCX 17 KB) [file 262_2024_3725_MOESM2_ESM.docx]

**Supplemental tables**

**Supplemental Table 1. Demographic characteristics**

| Characteristic | *n* = 92 | % |
| --- | --- | --- |
| Gender |  |  |
| Male: Female | 77: 15 | 83.7: 16.3 |
| Age |  |  |
| Median, range | 59, 36-88 |  |
| $\geq$ 60: $<$ 60 | 41: 51 | 44.6: 55.4 |
| Karnofsky performance scale |  |  |
| 50-70: 80-90 | 13: 79 | 14.1: 85.9 |
| Site |  |  |
| Upper: Middle: Lower | 4: 54: 34 | 4.3: 58.7: 13.0 |
| T stage |  |  |
| 1: 2: 3: 4 | 12: 11: 57: 12 | 13.0: 12.0: 62.0: 13.0 |
| N stage |  |  |
| 0: 1: 2: 3 | 40: 38: 9: 5 | 43.5: 41.3: 9.8: 5.4 |
| Stage |  |  |
| I: II: III: IV | 11: 27: 43: 11 | 12.0: 29.3: 46.7: 12.0 |
