# Supplementary figures and images for: Prognostic value of genomic mutation signature associated with immune microenvironment in southern Chinese patients with esophageal squamous cell carcinoma
Source: Cancer Immunol Immunother. 2024 Jun 4;73(8):141. doi: 10.1007/s00262-024-03725-2 (PMC11150228; doi:10.1007/s00262-024-03725-2)

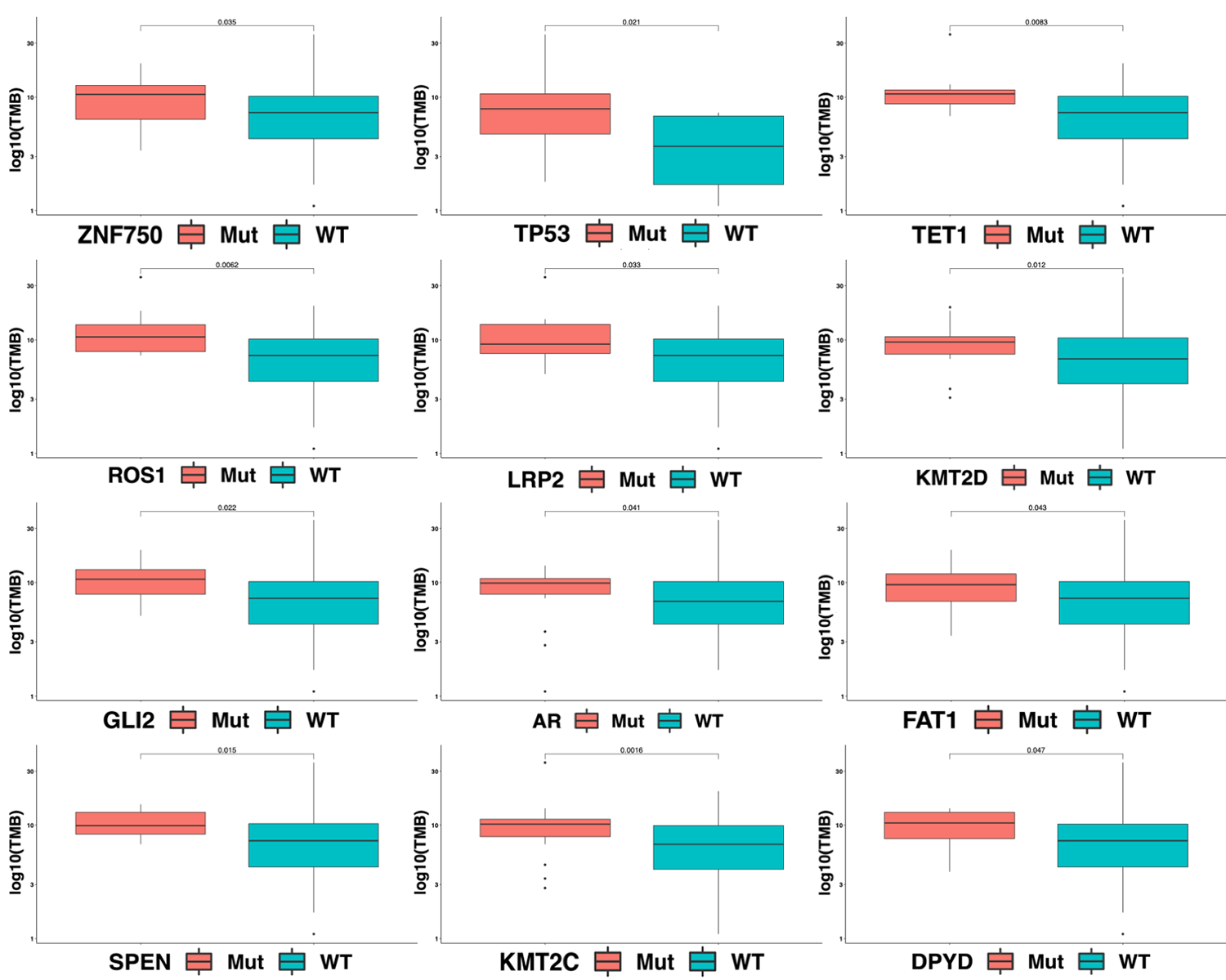

Supplement: Supplementary file 3 — Supplemental Fig. 1. The association between TMB and mutant genes (p < 0.05). (TIF 5297 KB) [file 262_2024_3725_MOESM3_ESM.tif]

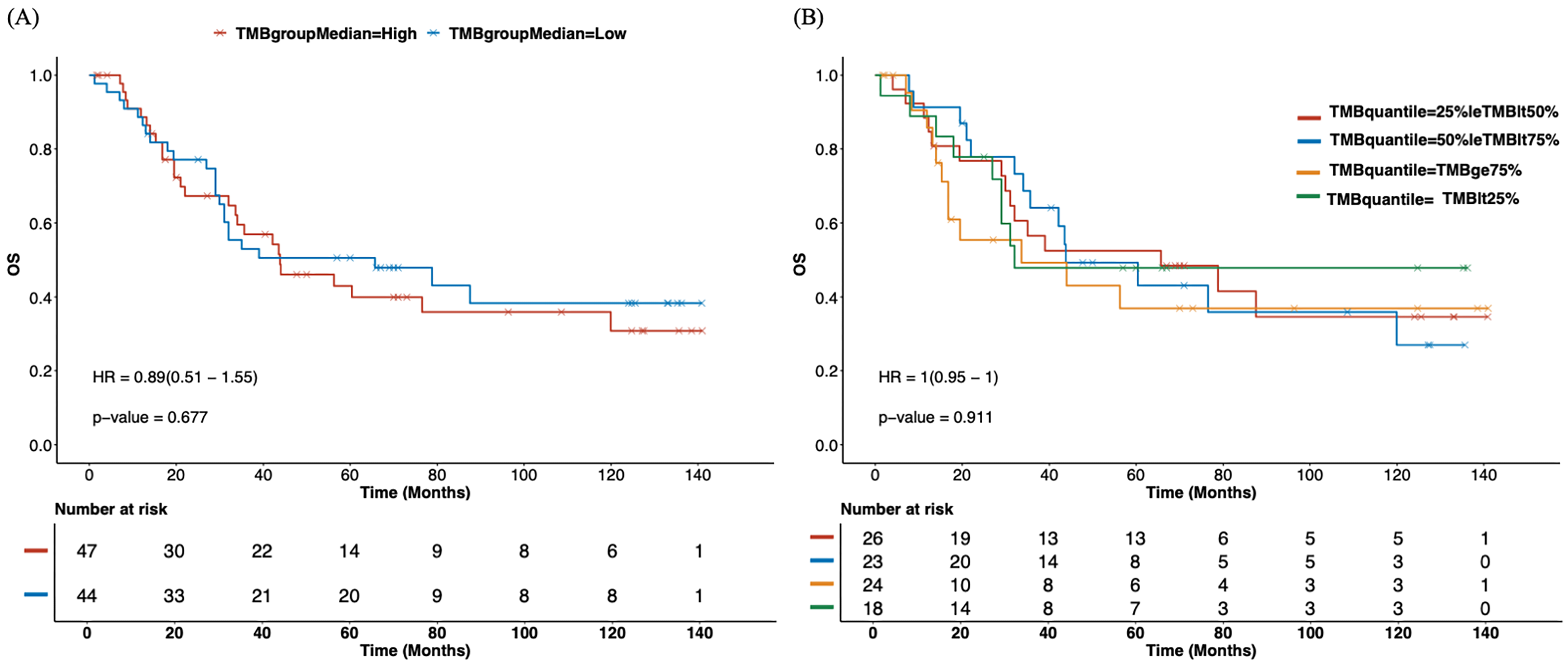

Supplement: Supplementary file 4 — Supplemental Fig. 2. The overall survival in the ESCC patients stratified by (A) the median value of TMB and (B) the quantile values of TMB (TIF 7147 KB) [file 262_2024_3725_MOESM4_ESM.tif]

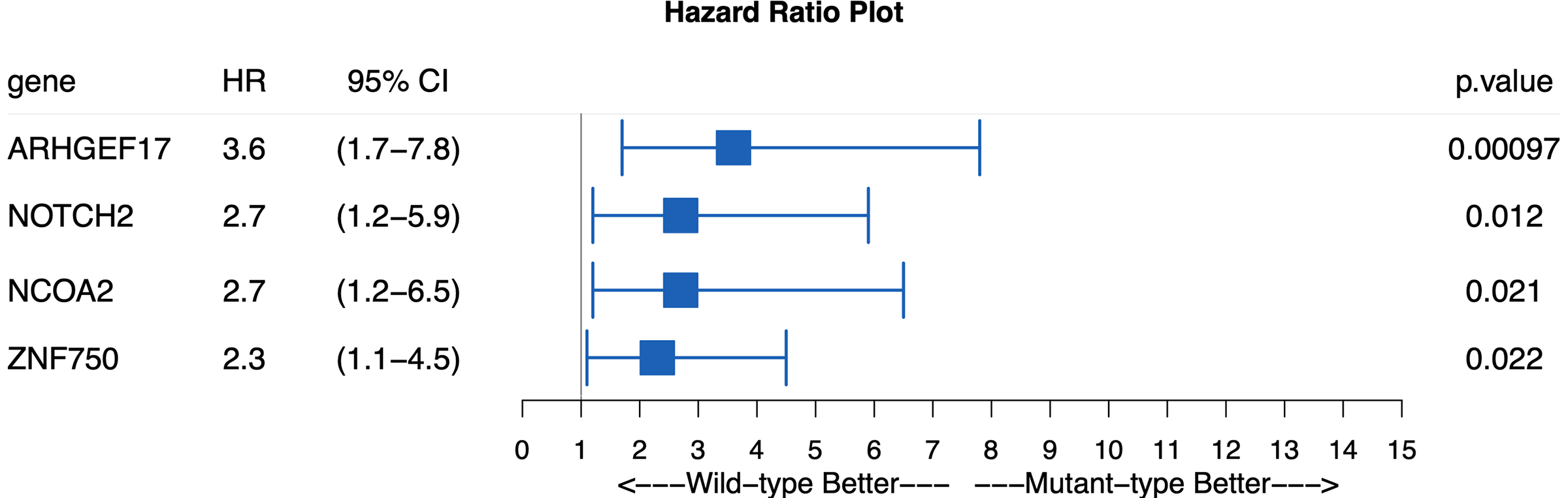

Supplement: Supplementary file 5 — Supplemental Fig. 3. Forest plot of the mutated genes in the 17MGR model associated with overall survival in ESCC (p < 0.1) (TIF 5388 KB) [file 262_2024_3725_MOESM5_ESM.tif]

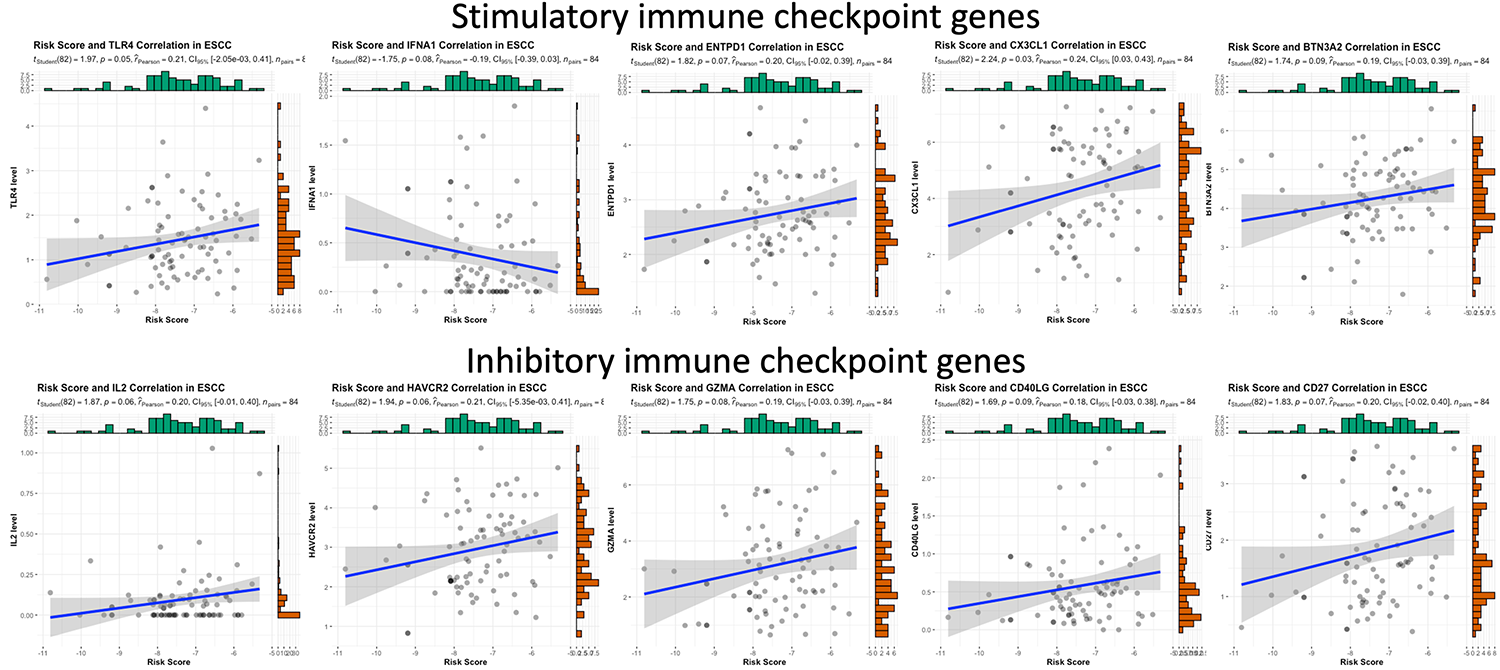

Supplement: Supplementary file 6 — Supplemental Fig. 4. The relationship between risk score based on the MGR signature and the level of stimulatory and inhibitory immune checkpoints (p < 0.1) (TIF 2970 KB) [file 262_2024_3725_MOESM6_ESM.tif]

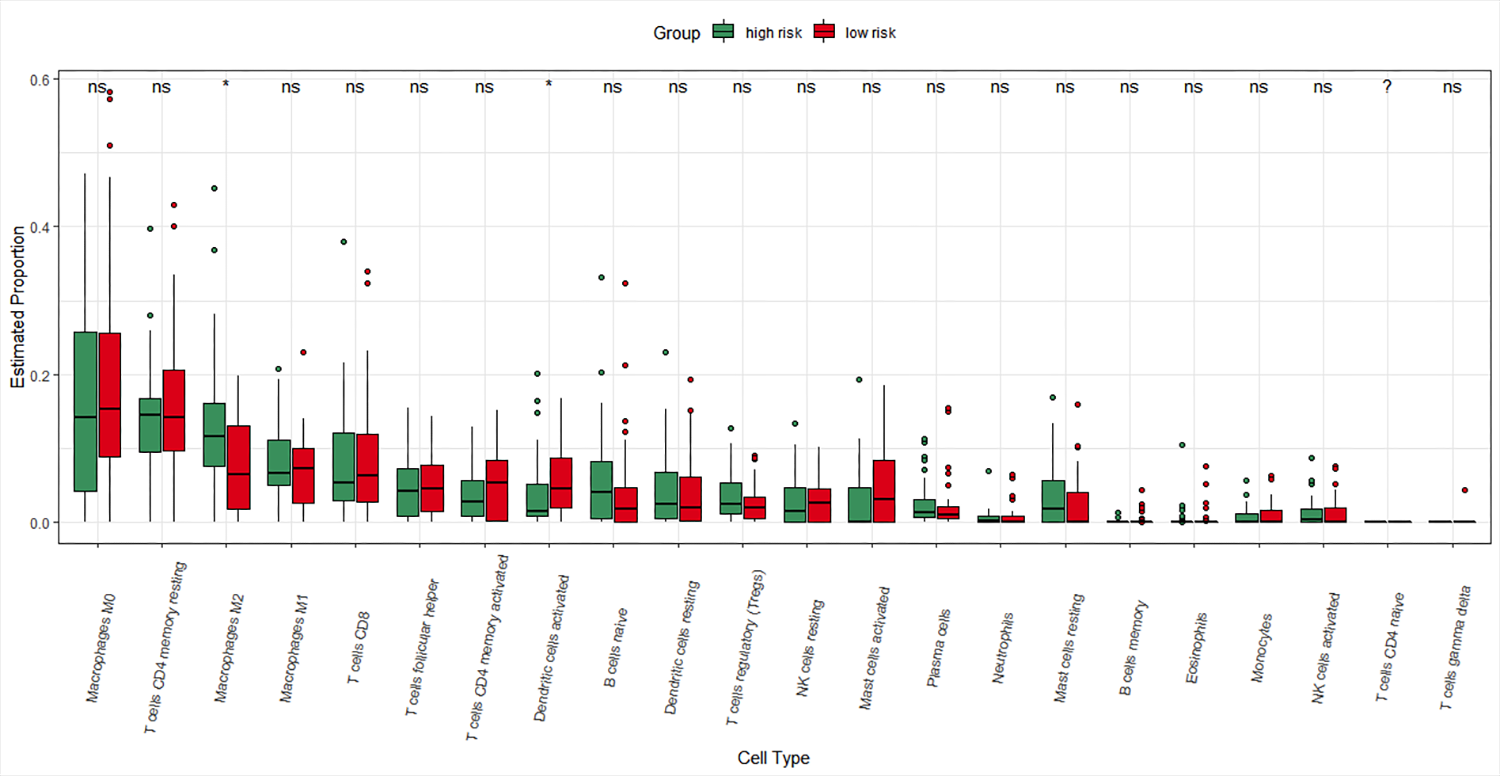

Supplement: Supplementary file 7 — Supplemental Fig. 5. The level of tumor-infiltrated immune cells estimated in the TCGA-ESCC cohort (arranged in descending order) (TIF 5128 KB) [file 262_2024_3725_MOESM7_ESM.tif]
